# Addressing the sublime scale of the microbial world: reconciling an appreciation of microbial diversity with the need to describe species

**DOI:** 10.1016/j.nmni.2021.100931

**Published:** 2021-08-13

**Authors:** I.C. Sutcliffe, R. Rosselló-Móra, M.E. Trujillo

**Affiliations:** 1) Faculty of Health and Life Sciences, Northumbria University, Newcastle upon Tyne, NE1 8ST, UK; 2) Grup de Microbiologia Marina, IMEDEA (CSIC-UIB), C/Miquel Marques 21, 07190, Esporles, Illes Balears, Spain; 3) Dpto. Microbiología y Genética, University of Salamanca, 37007, Salamanca, Spain

**Keywords:** Microbial culture, microbial diversity, phylogenomics, systematics, taxonomy

## Abstract

There are fewer than 20,000 prokaryotic species with validly published names, meaning >99% of a reasonable estimate of microbial diversity remains formally unnamed. Here we explore the damaging consequences of the current practice in which each new species is described in a standardized publication, most typically a ‘single strain species description’. This approach is both an impediment to scaling up progress in naming the microbial world and also a significant factor in the poor reputation of the discipline of microbial taxonomy. We conclude that significant changes in author habits are needed and make constructive suggestions as to how author practice should adapt.

Contemplating the scale of the microbial world is a sobering activity: even highly conservative estimates suggest ∼2 million prokaryotic species [1,2], while other estimates are orders of magnitude higher [3,4]. The continuing absence of an agreed species concept for prokaryotes notwithstanding [5,6], a pragmatic working estimate of not less than 10 million prokaryotic species can be deduced from the multi-million species richness of macroscopic eukaryotes and their attendant microbiomes before consideration is given to prokaryotes in the environment. Thus, it is not inappropriate to describe this diversity as *sublime*, a word with complex definitions that convey the mixture of awe, pleasure and even fear inspired by the grandeur of nature.

In accepting not less than 10^7^ species as being a reasonable estimation of prokaryotic diversity, it is appropriate to review what this means for the working methods of taxonomists. Accessing that diversity is clearly the first challenge – while estimates suggesting that only a few per cent of microbial diversity is culturable need to be revised upwards [7], it is evident that the vast majority of the recognized diversity of the microbial world is represented by ‘as yet uncultured’ taxa [2,6,8,9]. Regrettably, this has opened up a significant divide between those who prioritize culture-based characterization and classification versus those advocating sequence-based approaches to understanding microbial diversity (most notably using metagenome-assembled genomes). A compromise solution that could have resolved this impasse by allowing the use of genome sequence data as type [10] has been rejected by a vote of the members of the International Committee on Systematics of Prokaryotes [11], thereby widening the divide, which now threatens to become a full-blown schism. The development of a separate code of nomenclature for uncultivated taxa could provide an alternative solution [12,13], but there remains a clear and present danger of what has been described recently as a ‘wild west’ threatening to overwhelm the stable and regulated naming of prokaryotes [14].

The diversity of the prokaryotic world clearly begs the question: how will we ever name all these taxa, especially given the now entrenched requirement (if the name of a species is to be validly published) to cultivate type strains and deposit them in two publicly accessible culture collections in different countries, as detailed in Rule 30 of the International Code of Nomenclature of Prokaryotes (ICNP; [15]). In approximately two centuries since the naming of *Serratia marcescens* Bizio 1823 (Approved List 1980), fewer than 20,000 prokaryotic species names have been validly published (excluding synonyms), including ∼1200 cyanobacteria named under the Botanical Code (ICN, https://lpsn.dsmz.de/text/numbers), i.e. <0.2% of the estimated prokaryotic species diversity (Fig. 1). Currently, just over 1000 species names are validly published per annum [16], meaning that, even with a 10-fold increase in the current rate of description, it will take another thousand years to name the microbial world! Of course, it can be argued that not everything needs to be formally named, but it will still take another century to validly publish names for a ‘representative 1%’ at current rates (even assuming we can overcome the longstanding cultivation bias whereby currently ∼70% of validly named prokaryotic species belong to just four of ∼150 known phyla). These daunting numbers clearly suggest that new ways of working are urgently needed if we are to make significant progress in scaling up the naming of the microbial world.

**Fig. 1:**
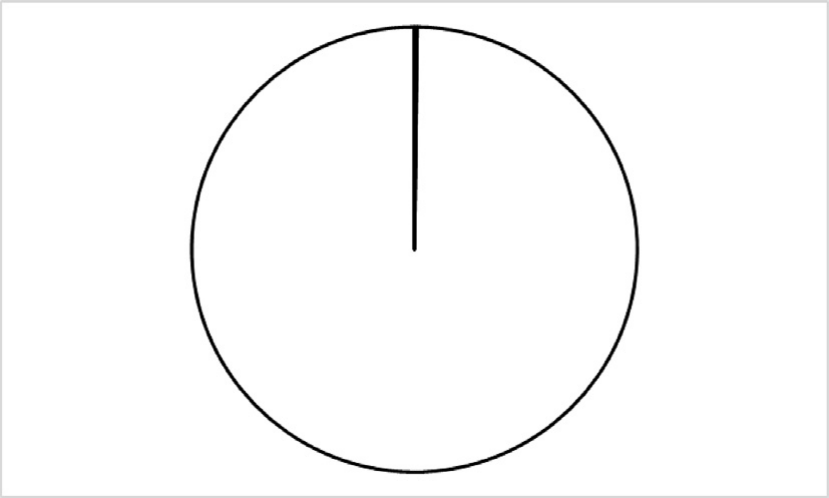
**The scale of the challenge.** Based on an estimated number of 10 million prokaryotic species, two centuries of microbial taxonomy have formally named only the black shaded area of the ‘pie of microbial life’.

One solution is to re-examine the way that novel taxa are described in scientific publications. The standard output from the work of traditional microbial taxonomists using the still pervasive ‘polyphasic’ taxonomic approach is the highly formulaic ‘taxonomic description’, manifest most commonly as single strain species descriptions (SSSD) in the *International Journal of Systematic and Evolutionary Microbiology* or a relatively small number of other journals [[17], [18], [19]]. Trujillo and Oren [16] aptly described these SSSD as ‘salami slicing’ taxonomic work into the ‘least publishable unit’. This unsustainable practice creates problems on a number of levels. The first is reputational: taxonomy has been bedevilled by a longstanding perception as a backwater ‘stamp collecting’ activity, memorably captured by Cowan’s comment that “Taxonomy is written by taxonomists for taxonomists; in this form the subject is so dull that few, if any, non-taxonomists are tempted to read it” [20]. This problem persists five decades later, with Oren and Garrity [18] observing that “Rosselló-Móra (2012) commented that “Even taxonomists might get bored when reading many of the ∼600–700 new descriptions of Bacteria and Archaea published each year.” [We] confirm that there is a great deal of truth in that comment. Indeed, formal taxonomic proposals are highly structured, standardized, textual descriptions of facts. They do not make exciting reading material.” The present authors have extensive experience as Editors of significant prokaryotic taxonomy journals and have edited many hundreds of taxonomic descriptions and can confirm that these criticisms are well justified: it is abundantly evident that the factory-farming of SSSD makes microbial taxonomists guilty of rendering their own outputs mundane and all too easily disparaged. In turn, this creates the secondary problem that this unenviable reputation inevitably makes microbial taxonomy seem an unattractive career choice when it comes to recruiting the next generation of scientists. As a possible solution, RRM, as Executive Editor of *Systematic and Applied Microbiology*, restricted the consideration of SSSDs only to very exceptional cases based on the interest of the strain itself and/or the quality of the descriptions. Unfortunately, this resulted instead in an avalanche of two strain species descriptions in which the isolates typically originated from the same sample, likely even the same enrichment plate, and still adhering to the same routine format for description.

The formulaic nature of many prokaryotic species descriptions derives in no small part from the ‘checklist’ repertoire of methodologies that are applied, many of which were regrettably fossilized by the 2010 “Notes on the characterization of prokaryote strains for taxonomic purposes” [21]. Criticisms have been raised elsewhere [[22], [23], [24]] as to why, in an age of genomic taxonomy, routine taxonomic descriptions are still packed with data from batteries of irrelevant phenotypic and chemotaxonomic tests that add little insight into the biology of the taxon under study. Cowan was again prescient here, scathingly dismissing (albeit in a slightly different context) an over-reliance on a few phenotypic characters, which “By virtue of the length of time for which they have been used […] have assumed an importance out of all proportion to their usefulness.” [25] The contemporary equivalent to the tests he highlighted (such as gelatin liquefaction) is what can be described as the ‘cystine arylamidase conundrum’: this and multiple other enzyme activities are often reported in taxonomic studies because they can be assayed using popular miniaturized diagnostic kits, despite there being a scant appreciation of whether these enzyme activities are of any particular relevance to the biology of the taxon under study. Many formal descriptions (‘protologues’, see below) even contain antibiotic sensitivity data, which is particularly problematic for SSSD (i.e., the majority) given that antibiotic susceptibility can so obviously vary within a population of strains. This illustrates well that many classical phenotypic tests are performed not so much for their discriminatory value or information content but simply because of their availability and familiarity. These habits are perpetuated both by authors wanting to stay within their comfort zones, turning the handle to crank out more papers and by the misplaced insistence of overly conservative editors and peer reviewers that such tests are ‘mandatory’ (despite this dogmatism contradicting Principle 1.4 of the ICNP). A further problem with this situation is the consumption of valuable resources. These include not only the labour and direct costs expended in conducting such studies (including the repeat analysis of reference strains) and writing them up, but also in their peer review and editorial handling. Is it reasonable to expect the same approach to be sustainable for the next 100,000 species descriptions, never mind the next million?

The gravity of this situation demands constructive and rapidly implementable solutions, driven by the application of new concepts, frameworks and taxonomies. At the heart of the problem is that the “one colony – one species – one article” publication model [16] is unsustainable. Even within that approach, a first step must be for authors to explain better in their papers why a particular taxon (or the source it came from) is of interest to study, thereby improving the significance of their work and raising interest in it. Several journal’s Instructions for Authors already request this [26], including the *International Journal of Systematic and Evolutionary Microbiology* article template, although such requests seem to be largely ignored. Most importantly, authors habits need to change to embrace working on a larger scale. In the first instance, this should involve avoiding salami slicing by instead bundling the descriptions of several taxa into single papers wherein an overarching theme such as ecology, metabolism or isolation source/methodology can be used to connect the taxa being described. Recent examples are the description of six *Paenibacillus* species obtained using the same isolation strategy [27], nine *Winogradskyella* species from a high throughput cultivation strategy [28] and the effective publication of names of multiple taxa from microbiome studies [29,30]. Improving the contextualization of work in this way will hopefully make taxonomic studies of more general interest and thus help reconnect the work with (and encourage the participation of) a wider audience from outside of the specialized systematics community.

Taking this approach still further, taxonomists need to consider genuinely high-throughput approaches that will allow authors to name much larger numbers of taxa in single reports. Progress is being made here through the application of ‘culturomics’ and other novel strategies [[31], [32], [33], [34], [35]]. Specifically, the integration of MALDI-TOF mass spectrometry as a high-throughput phenotypic screen with the sequence-based assessment of sample diversity needs to be encouraged. Compared to ‘traditional’ phenotypic approaches, the MALDI-TOF MS screening approach offers the significant advantage of generating ‘portable’ data that can be added into spectral databases, although it is regrettable that there are not yet available any ‘open research’ platforms for this comparable to the way that the International Nucleotide Sequence Database Collaboration provides access to sequence data (http://www.insdc.org/). Nevertheless, MALDI is poised to move from primarily clinical applications and microbiome studies into broader applications in microbial ecology [31,35,36]. Such approaches will no doubt be well complemented by other novel approaches to culturing previously uncultivated taxa [33,[37], [38], [39]] and the further development of high-throughput ‘next-generation physiology’ [40] and ‘metabolomic taxonomy’ fingerprinting approaches.

## Working at high throughput and larger scale will bring new challenges

One is that such studies require the generation of huge numbers of Latin binomial species names, which remain of fundamental importance to an effective and functional biological nomenclature [41]. A significant step forward in scaling up the formulation of Latin names was recently made through the release of the ‘Great Automatic Nomenclator’ (GAN, https://github.com/telatin/gan), which allows the combination of prefixes with appropriate endings to generate grammatically accurate Latin names, with etymologies [41]. Although GAN will need refinement to improve its functionality and user-friendliness, it is a powerful proof-of-concept that Latin binomial names can be generated by the million. Notably, GAN was applied to the generation of names for >650 *Candidatus* taxa identified as metagenome-assembled genomes in the chicken microbiome [30], thereby in a single study increasing the number of well-formed *Candidatus* names by >50%. An alternative (that could run in parallel) would be to generate a large repository of unused correctly formulated names and epithets, from which authors could pick and use in their descriptions.

A second challenge in accelerating the rate at which prokaryotic species are named comes with the formulation of the accompanying protologues, i.e., that part of a publication which is set aside to describe a taxon in accordance with the requirements of Rule 27 of the ICNP. (It should be noted that the term protologue is not itself used in the ICNP). As noted above, conventional protologues are often dominated by large amounts of phenotypic data of questionable relevance, such as miniaturized kit results. The generation of very large numbers of conventional protologues is undoubtedly impractical and arguably unnecessary. Renner [42] has persuasively argued that taxonomists should place renewed emphasis on *diagnosis* rather than *description,* i.e., a focus on those characters that allow the unambiguous identification of the taxon if it is encountered again. This approach is greatly facilitated by the comprehensive adoption of whole-genome sequencing in prokaryotic taxonomy [6,43], which itself provides a welcome example of the willingness of taxonomists to adopt novel technologies. Examples are now beginning to accumulate where protologues are limited to the name and etymological information, type strain details, brief descriptions of relevant information such as isolation source, culture conditions, sequence accession numbers and statements defining how the taxon is delineated phylogenomically from analysis of the type strain genome (for examples, see Table 1 of Gilroy et al. [30]). Generating and curating this information may be facilitated by new tools such as protologger (http://protologger.de/), which also integrates GAN [44].

Accelerating this trend towards *diagnosis* should encourage the taxonomic community to reappraise the expectation that all prokaryotic species are described in full papers. Just as reports of genome sequences have migrated from papers in high prestige journals (Nature, Science, etc.) to ‘genome announcements’ (or even simply accession numbers), short-form article types are needed for prokaryotic species descriptions (currently well illustrated by the *New Microbes and New Infections* New Species Announcement article type). Ultimately, it may even prove possible that novel species are announced not by the publication of published descriptions but instead via entries into a database [24,45,46], although our attempt to establish the Digital Protologue Database as a precursor to such a database was regrettably short-lived [46,47]. If database entries ultimately substitute for unnecessary taxonomic descriptions in journals, author credit for describing new taxa could be derived from the database ‘microattribution’, underpinned via citable persistent identifiers such as DOI [48]. The prospect (indeed necessity) of naming microbial species at rates of at least an order of magnitude greater than currently suggests that revisiting this database-driven approach is highly desirable, perhaps even essential, although it would likely mean amendment/clarification as to what constitutes an acceptable forum for publication in Rule 25 of the ICNP. Such a database could potentially also serve to record additional members added to a species since these can often remain unpublished following the publication of the original description.

## Working at high throughput and larger scale will also bring new opportunities

The above minimal diagnosis/digital protologue approach to naming prokaryotes seems entirely appropriate given that when a species is just one in 10^7^ in the biosphere, not all will be of any great individual interest. For example, if a genus already contains multiple members, it becomes increasingly hard to find something genuinely novel to report about the next members to be isolated when using the current portfolio of standard taxonomic tests. In this regard, we have seen, disappointingly, many examples of genomes sequence data being significantly under-utilized in taxonomic papers, sometimes being used solely to obtain Average Nucleotide Identity/digital DNA:DNA hybridization metrics and G+C content.

Removing the emphasis on SSSD as the basic unit for building Curriculum Vitae will also allow redirection of resources (both time and expenditure) towards greater synthesis, such as systematic overviews of genera (e.g., [49]) and higher taxa (for examples see [[50], [51], [52]]. The consequently accelerated naming of taxa and placing of those names within a robust phylogenomic context will allow the retrospective selection of representative taxa for more in-depth characterization, including targeted investigation of properties deduced from an interrogation of the genomes (such as secondary metabolite biosynthesis capabilities, metabolic pathways, or features deduced from comparative genomics to be relevant to functional ecology and biogeography), with inter-connected databases supporting these activities [45]. Inclusion of *Candidatus* taxa and MAGs in large scale phylogenomic studies will also highlight those branches of the microbial world currently lacking cultured representative, such that genomic analyses can be used to inform the targeted development of methods to enable isolation and culturing [[37], [38], [39]]. A retrospective approach may also revitalize chemotaxonomy, transforming it from its current laborious misapplication in the characterization of species individually into compelling surveys across higher taxonomic levels (i.e., of genera, families, etc.), with state-of-the-art analytical chemistry techniques applied by appropriate experts. Indeed, this would be a return to the type of comparative studies performed at the advent of chemotaxonomy (e.g., [[53], [54], [55]]) and could be complemented by ‘*in silico* chemotaxonomy’ approaches, wherein genomic data is interrogated to understand the distribution of biosynthetic pathways for key markers [22,56].

## Concluding comments

**Fig. 2:**
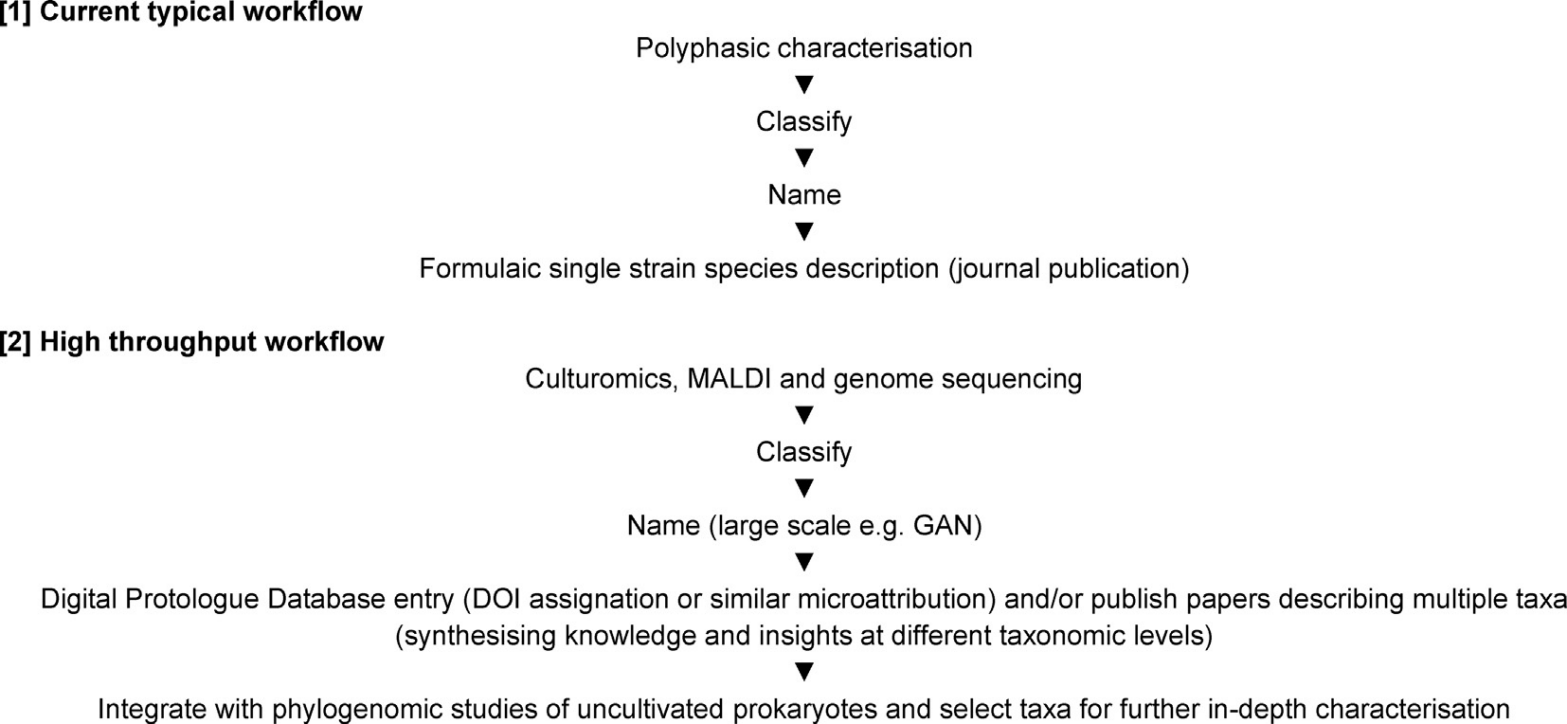
**Current versus proposed approaches to describing the microbial world.** With the status quo ([1]), ∼1000 species are named per annum. In a ‘high throughput’ approach [2], it may become feasible to target the naming of 10,000+ species per annum.

Nearly a decade ago, Sutcliffe et al. [22] highlighted that “a significant reappraisal of the procedures used to describe novel prokaryotic taxa is needed, including the likely introduction of new publication formats.” It is increasingly self-evident that the ‘one species – one paper’ publication model currently supporting careers in microbial taxonomy is unsustainable, given the sublime scale of the microbial world. It is hoped that the suggestions above will encourage the wider community of microbial taxonomists to embrace new ways of working (Fig. 2) and inspire greater ambition in the scale at which taxonomic studies are performed. In these ways, taxonomic studies can be used to underpin and drive novel lines of enquiry, generating papers of wider interest that should help counter the above-mentioned poor reputation of taxonomy as a ‘stamp collecting’ activity and promote it as an engaging field attractive to early-career scientists. Such studies will highlight the relevance of taxonomic work and its significance as a vital underpinning for microbiological research to address global challenges [57].

Despite the challenges of securing funding for taxonomic work, community efforts to develop and sustain a Digital Protologue Database must be renewed to support this new model. We encourage the systematics community to explore the suggestions presented here and invite the International Committee on Systematics of Prokaryotes to consider how best to drive community engagement with these proposals. However, regrettably, it is also clear that there is an innate conservatism in the taxonomic community that must be overcome: as John Maynard Keynes [58] wrote in the *Preface to The General Theory of Employment, Interest and Money*, “The difficulty lies, not in the new ideas, but in escaping from the old ones.”

## Author contributions

Iain C. Sutcliffe, writing - original draft; Ramon Rosselló-Móra, writing - review and editing; Martha Trujillo, writing - review and editing.

## Transparency declaration

IS is an Editor of Antonie van Leeuwenhoek (formerly Editor-in-Chief); RRM is an Executive Editor of Systematic & Applied Microbiology; MET is Editor-in-Chief of International Journal of Systematic and Evolutionary Microbiology.
